# The Effects of Short-Duration Ischemic Preconditioning on Horizontal and Vertical Jump Performance in Male and Female Track and Field Jumpers

**DOI:** 10.3390/jfmk10030265

**Published:** 2025-07-14

**Authors:** Varvara Nektaria Gkari, Athanasios Tsoukos, Nikolaos Aspradakis, Gregory C. Bogdanis

**Affiliations:** School of P.E. and Sport Science, National and Kapodistrian University of Athens, 17237 Athens, Greece; vanagkari@phed.uoa.gr (V.N.G.); nikosaspr@phed.uoa.gr (N.A.); gbogdanis@phed.uoa.gr (G.C.B.)

**Keywords:** hopping, drop jump, triple jump, long jump, blood flow

## Abstract

**Background**: Ischemic preconditioning (IPC) is a non-invasive, time-efficient strategy that has been shown to acutely enhance athletic performance. The present study examined the effects of 5 min of IPC on vertical and horizontal jump performance. A secondary aim was to explore the associations between outcomes of the 5-Hop (5-H) test and drop jump performance, in order to provide further evidence supporting the validity of the 5-H test for assessing reactive strength characteristics in trained jumpers. **Methods**: Twelve trained track and field jumpers (nine males, three females, age: 23.2 ± 2.9 years; height: 1.76 ± 0.07 m; body mass: 71.5 ± 8.0 kg) completed two conditions: an IPC condition applied to one leg and a control condition applied to the contralateral leg. In the first week, one leg was assigned to IPC and the other to the control condition, while in the second week, the conditions for each leg were reversed. Vertical single-leg performance was evaluated by drop jump (DJ) height, ground contact time, and reactive strength index (RSI). Horizontal jump performance was assessed by a five-hop (5-H) test during which total distance (TD), total time (TT), and reactive hopping index (RHI) were obtained. **Results**: Compared to the control condition, IPC enhanced DJ height (+ 3.6%) and RSI (+ 7.8%) (*p* < 0.05, g = 0.16 and 0.32, respectively) and reduced contact time (−4.4% *p* < 0.05, g = 0.41). Also, IPC resulted in significant improvements in TD (+ 4.1%) and RHI (+ 3.9%) during the 5-H test (*p* < 0.05, g = 0.32 and 0.42, respectively), while TT remained unchanged. **Conclusions**: A single cycle of IPC acutely improved vertical and horizontal jump performance and reactive strength indices in trained jumpers. These findings support the use of IPC as a practical, time-efficient method to enhance neuromuscular performance in explosive tasks.

## 1. Introduction

Optimizing athletic performance is a well-established, long-term objective for both coaches and athletes. Active warm-up is a commonly employed method to enhance performance, as its mechanisms—such as increased muscle temperature, metabolic adaptations, and neurophysiological changes (i.e., enhanced phosphocreatine resynthesis, elevated enzymatic activity related to ATP production, improved nerve conduction velocity, and increased motor unit recruitment efficiency)—prepare the body for subsequent athletic activity [1,2,3]. In recent years, advances in sports science research have led to the development of several interventional methods—collectively known as “preconditioning techniques”—such as post-activation performance enhancement (PAPE), passive heat maintenance, and dynamic warm-ups, all of which aim to further enhance athletic performance [4,5]. One such method is ischemic preconditioning (IPC), first identified in 1986 for clinical purposes [6]. IPC is a non-invasive, time-efficient technique that appears to confer protection against ischemia–reperfusion injury, which primarily results from oxidative stress, calcium overload, and inflammatory responses following prolonged ischemia and reperfusion [7,8,9]. IPC typically involves a series of brief cycles of complete blood flow restriction in one or more muscle groups (either upper or lower limbs), using blood flow restriction cuffs that are inflated to a certain pressure. Each occlusion phase is followed by a reperfusion cycle, during which the cuffs are deflated to reestablish blood circulation [10,11,12]. The duration and number of occlusion cycles vary across studies, with 3–4 cycles of 5 min occlusion followed by 5 min reperfusion being the most commonly used protocol [12,13,14].

Physiological mechanisms underlying IPC are not fully elucidated due to their complexity and the multitude of performance-influencing variables. However, IPC has been shown to acutely enhance metabolic efficiency by attenuating ATP and glycogen depletion and reducing lactate accumulation during ischemic conditions, while simultaneously improving skeletal muscle perfusion through vasodilation [6]. Specifically, increased nitric oxide (NO) bioavailability is suggested to be one of the primary mechanisms of IPC-induced performance enhancement, as NO is a potent vasodilator responsible for vessel dilation during reperfusion, which enhances blood flow and oxygen delivery to active muscles [13]. Additionally, some studies suggest that IPC improves neuromuscular efficiency by attenuating the activity of Type III and IV muscle afferents, which are associated with fatigue [13,15,16,17]. Furthermore, IPC has been noted to elicit effects similar to the Post-Activation Performance Enhancement (PAPE), which is known to immediately boost athletic performance [5,14].

Several studies have explored the effects of IPC on athletic performance, with outcomes ranging from positive to neutral. IPC has demonstrated benefits in strength-related tests involving resistance exercises. For instance, Guilherme Da Silva Telles et al. [1] reported that ischemic preconditioning (IPC), applied as four cycles of 5 min ischemia followed by 5 min reperfusion, increased the number of repetitions performed against 80% of one-repetition maximum (1RM) in the leg press exercise by 25%, with improvements reaching up to 50% across three sets. These gains were observed in comparison to various warm-up protocols including SHAM, specific warm-up, aerobic exercise, and active stretching. Additionally, in the bench press exercise, IPC led to a 41.8% increase in repetitions in the first set and a 43.6% increase in the second set compared to the specific warm-up, as well as a 46.8% increase in the third set compared to SHAM. These findings underscore the potent effect of IPC on enhancing muscular endurance across multiple sets. [1]. Salagas et al. (2022) observed similar improvements in the bench press exercise using a single 5 min ischemia cycle with 5 min of reperfusion [5]. Their study reported a 7.6% increase in total repetitions (*p* = 0.019, g = 0.42). and a significant enhancement in average mean bar velocity (AMV), which was 9.0% higher in the ischemic preconditioning (IPC) condition compared with control during the first set (*p* < 0.01, g = 0.77). This elevated AMV was maintained in the second and third sets, showing increases of 7.0% and 6.6%, respectively. Furthermore, for average peak velocity (APV), IPC resulted in values 7.8% higher than control across all sets (*p* = 0.044, g = 0.40), demonstrating superior neuromuscular performance compared to both post-activation performance enhancement (PAPE) and control conditions. Additionally, a recent meta-analysis concluded that IPC significantly improves one-repetition maximum (1RM) performance in healthy adults, with a mean difference of 1.38 kg compared to placebo (95% CI: 1.05–1.72, *p* < 0.00001) [18]. IPC also appears effective in enhancing aerobic and anaerobic performance. For example, VO_2_max increased from 56.8 ± 6.8 to 58.4 ± 6.2 mL·min^−1^·kg^−1^ in a sample of 15 participants following IPC (*p* = 0.003, Cohen’s d ≈ 0.25), indicating a small but statistically significant effect [19]. Additionally, compared to placebo, IPC led to substantial improvements in repeated sprint cycling performance, with peak power output increases of 2.4% ± 2.2% in sprint 1 (89% likely, small effect), 2.6% ± 2.7% in sprint 2 (87% likely, small effect), and 3.7% ± 2.4% in sprint 3 (97% very likely, small effect) [20]. IPC has also been shown to improve vertical jump performance (Cohen’s d = 0.50) [14].

Track and field athletes, particularly jumpers, perform numerous vertical and horizontal jumps during training and competition. In jumping events, outcomes are often decided by minimal margins; therefore, even small improvements in performance may confer a competitive advantage. For instance, in elite long jump competitions, final rankings are frequently separated by differences of less than 5 cm [21]. As a result, identifying training or preconditioning strategies that can elicit even small enhancements in explosive power or take-off efficiency may prove critical for success at the highest levels of performance. Although IPC has shown promise in improving vertical jump performance, with effect sizes ranging from moderate to large (Cohen’s d = 0.50 to 1.16) [14,22], research on specific explosive performance indices and its effects on horizontal jump performance is lacking. Notably, no studies have assessed IPC’s impact on continuous horizontal plyometric exercises, such as those relevant to triple jump performance. Additionally, most studies on IPC employ protocols with 3–4 cycles of 5 min ischemia and reperfusion, which may not align with the time-efficiency goals of modern athletic training and competition. Examining shorter IPC protocols, such as a single 5 min ischemia cycle followed by 5 min of reperfusion, may offer a practical and efficient alternative without compromising performance benefits. Furthermore, to our knowledge, only one study has examined a single 5 min IPC cycle followed by reperfusion, reporting favorable effects on resistance exercise performance [5].

Therefore, the aim of this study was to investigate the effects of a single 5 min IPC cycle with a 5 min reperfusion period on the performance of single-leg drop jump (DJ) and five repeated hops on the same leg (5-H) with two steps, in male and female track and field jumpers. The single-leg DJ was assessed using three parameters: jump height, ground contact time, and reactive strength index (RSI). The 5-H test performance included three key parameters: total hopping distance (TD), total ground contact and flight time across all hops (TT), and mean horizontal velocity, calculated as total distance divided by total time. This velocity measure was expressed as the reactive hopping index (RHI), an indicator of explosive horizontal reactive strength during repeated unilateral hopping [23,24]. A secondary aim was to explore the associations between outcomes of the 5-Hop (5-H) test and drop jump performance, in order to provide further evidence supporting the validity of the 5-H test for assessing reactive strength characteristics in trained jumpers. Statistical analyses were conducted to compare performance between the dominant and non-dominant leg, as well as between the IPC and control (no IPC) conditions.

## 2. Materials and Methods

### 2.1. Participants

Twelve trained long- and triple-jumpers (nine males, three females, age: 23.2 ± 2.9 years; height: 1.76 ± 0.07 m; body mass: 71.5 ± 8.0 kg; BMI: 23.1 ± 2.4 kg·m^−2^) voluntarily participated in this study. The participants in our study had an average of 8.6 ± 4.5 years of systematic training background in long- and triple-jump, and, therefore, were very familiar with the horizontal and vertical jumping exercises used in this study. As such, they possessed a high level of technical proficiency, which likely minimized inter-trial variability in terms of technical execution. None of the participants had a history of injury in the past year and had not used any medication during the experimental period. Prior to participation, they were informed in detail about the study’s purpose, procedures and potential risks, and provided written informed consent. They were also informed of their right to withdraw from the study at any point without penalty. Participants were recruited via convenience sampling. All procedures were approved by the Bioethics Committee of the School of Physical Education and Sports Science of Athens, Greece (Approval no. 1508/19-04-2023), and conducted in accordance with ethical standards.

### 2.2. Research Design

**Figure 1:**
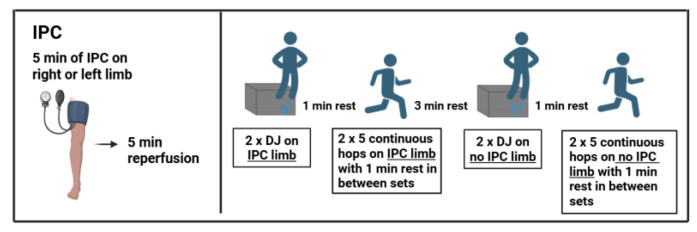
Schematic representation of the experimental protocol. IPC: Ischemic preconditioning; DJ: drop jump.

A randomized, counterbalanced, repeated-measures design was employed for this study. Prior to the main testing, participants attended a familiarization session, during which their height, body mass and body mass index (BMI) were recorded, and they were introduced to the ischemic preconditioning (IPC) protocol. The experimental procedure spanned two consecutive weeks. Each week, participants completed both an experimental trial (with IPC) on one leg and a control condition (without IPC) on the contralateral leg. Both conditions followed an identical sequence of exercises: two repetitions of single-leg DJ followed by two repetitions of the 5-H test (Figure 1). The order of leg assignment (experimental vs. control) was randomized and counterbalanced across participants. In the first week, one leg was assigned to the experimental condition and the other to the control condition; in the second week, the conditions for each leg were reversed. All testing sessions were conducted one week apart and at the same time of the day to minimize diurnal variations and environmental influences (e.g., temperature, sunlight). All testing was performed on a standard outdoor track and field surface between 10:00 and 13:00, under ambient environmental conditions (mean temperature: 20–25 °C; relative humidity: 50–60%). Statistical analyses were conducted to compare performance between the dominant and non-dominant leg, as well as between the IPC and control (no IPC) conditions. The dominant leg was defined as the one used for take-off during the athletes primary jumping event (triple or long jump), while the opposite leg was considered the non-dominant leg. The selection of the DJ and 5-H test in this study was based on the specificity of movement demands relevant to horizontal jump events (i.e., long jump and triple jump), which rely heavily on reactive strength—the ability to rapidly transition from eccentric to concentric muscle action during the stretch-shortening cycle (SSC) [25,26,27]. Both DJ and 5-H are fast SSC tests and better reflect the neuromuscular and biomechanical characteristics of take-off actions in horizontal jumping disciplines. In contrast, the countermovement jump (CMJ), which involves a slower SSC, is considered less specific for evaluating reactive strength in this context.

### 2.3. Familiarization Session

Before the main experimental phase, all participants attended a preliminary session at the School of Physical Education and Sports Science of Athens. During this session, body mass was measured to the nearest 0.1 kg using a digital scale (Salus, Milan, Italy) with participants wearing minimal clothing, and standing height was assessed using a stadiometer attached to the same device, with participants barefoot. Body mass index (BMI) was subsequently calculated from these anthropometric measurements. Following the assessment, participants were escorted to the university’s track and field facility for familiarization with the experimental procedures. After completing a standardized 5 min warm-up consisting of low-intensity running and dynamic stretching, participants underwent a demonstration of the ischemic preconditioning protocol. IPC was applied at 250 mmHg for five minutes on either the right or left leg, followed by five minutes of reperfusion, to acquaint participants with the sensation and procedure of the intervention. Subsequently, participants performed three repetitions of the DJ exercise and 3 continuous horizontal hops on each leg to familiarize themselves with the experimental procedures, as all athletes were already proficient in the required movement techniques.

### 2.4. Standardized Warm-Up

Prior to each experimental session, participants completed a standardized 20 min warm-up consisting of an 8 min low-intensity run, followed by whole-body dynamic stretching and movement-specific drills replicating those used during the testing sessions. This was supplemented with some basic running and jumping exercises [28]. Immediately after the warm-up, participants performed the experimental condition, followed by the control condition.

### 2.5. IPC Protocol

Following the standardized warm-up, participants assumed a supine position on a padded examination mattress. A pneumatic blood flow restriction cuff equipped with a manual manometer (FitCuffs^®^, Odder, Denmark) was applied to the proximal portion of the thigh on the leg designated for the IPC condition. The cuff was then inflated to a pressure of 215 ± 21 mmHg to induce arterial occlusion for five minutes. To verify the complete cessation of blood flow, a Doppler ultrasound device (Sonotrax lite, Edan, Shenzhen, China) [29,30] was positioned over the posterior tibial artery on the medial aspect of the ankle. The absence of arterial flow was confirmed individually for each participant, and the pressure required for full occlusion was recorded to ensure consistency in subsequent sessions. After a five-minute occlusion phase, the cuff was fully deflated to allow reperfusion. During the ensuing five-minute reperfusion period, participants remained in the supine position.

### 2.6. Drop Jump Test

Immediately following the reperfusion phase, participants proceeded to perform the single-leg drop jumps (DJs) from a 20 cm high box. Two DJs were executed using the same leg that was subjected to the IPC protocol, with a 30 s rest interval between attempts. Each trial began from a single-leg upright stance on the box. Participants then stepped off, performed a countermovement to a self-selected depth upon ground contact, and immediately executed a maximal vertical jump, landing again on the same leg [31]. Throughout the entire movement, participants kept their hands on their hips to eliminate arm swing influence. Prior to each trial, participants received standardized verbal instructions to minimize ground contact time and to jump as high as possible. Jump performance was assessed using an optical measuring system (OptojumpNext, Microgate, Bolzano, Italy) [32], which recorded flight and contact times through its integrated sensor bars [33,34]. The primary outcome measures included jump height (cm), ground contact time (ms) and reactive strength index (RSI, m∙s^−1^). Both jumps were recorded, and the average of the two was used for further analyses.

### 2.7. Five Continuous Hop Test

After a one-minute rest, participants performed two repetitions of the five continuous hop (5-H) test, with a one-minute rest between trials. Athletes initiated the task by placing their take-off foot forward and executing two preparatory steps. The touchdown of the second step marked the starting point of the 5-H sequence. The hopping technique followed the guidelines described by Hay (1992), emphasizing optimal biomechanics. Specifically, the take-off leg was aligned vertically beneath the centre of gravity to maximize vertical lift [35]. Participants were allowed to use either a double-arm swing (where both arms were driven forward) or a single-arm technique, which mimics the natural arm motion of sprinting, according to their preference [35]. During flight, the take-off leg moved from a trailing to a forward position in preparation for landing, aiding in trunk stabilization and minimizing backward rotation [36]. Some athletes employed the hitch-kick technique, wherein the free leg is swung forward while the take-off leg flexes, helping to control rotational inertia and maintain trunk stability [37]. An “active landing” technique was used throughout, in which the landing foot executed a backward “pawing” motion to reduce braking forces and facilitate smooth ground contact [38]. Performance during the 5-H task was recorded using a high-speed digital camera (Exilim ZR-1000, Casio Computer Co, Ltd., Tokyo, Japan), which was positioned perpendicular to the mid-runway, 15 m away from the participants [28]. The camera operated at a sampling frequency of 120 frames per second [39]. The recordings were later analysed using Kinovea video analysis software (v.0.8.15) to determine total hopping distance and total duration. Mean horizontal velocity was then calculated based on these variables. For calibration, two ground markers spaced 6 m apart were used, and a measuring tape was also placed on the runway for real-time total distance evaluation. The starting point for the measurement of 5-H distance was defined as the toe contact of the second preparatory step, and the endpoint was the toe contact of the final (fifth) hop. After a three-minute recovery period, participants repeated the same exercise protocol under the control condition, performing the task with the contralateral leg, which had not been subjected to IPC. In this test, we assessed total hopping distance (TD), total time (TT), and mean horizontal velocity, calculated as the ratio of distance to time. These variables were used to compute reactive hopping index (RHI), an index of explosive horizontal reactive strength during repeated unilateral hopping [23,24].

$$\mathrm{Reactive}\;\mathrm{Hopping}\;\mathrm{Index}=\frac{total\;hopping\;distance\;(m)}{total\;ground\;contact\;and\;flight\;time\;across\;all\;hops\;(s)}$$

The reliability statistics for the 5-H test are presented in Table 1. The validity of a similar test has been demonstrated in previous studies [23]. Additionally, Table 2 shows a strong correlation between the 5-H test and drop jump performance (r = 0.66–0.94), further supporting its validity.

### 2.8. Statistical Analysis

All variables are presented as means ± standard deviations. Statistical analyses were performed using IBM SPSS Statistics (version 28.0; IBM Corp., Armonk, NY, USA). Values from the IPC and control leg, as well as from the dominant and the non-dominant leg, were averaged accordingly. To assess potential differences across conditions, a two-way analysis of variance (ANOVA) was conducted with two factors: condition (IPC vs. control) and leg dominance (Dominant vs. Non-Dominant). When a significant main effect or interaction was observed, Tukey’s post hoc test was applied. Effect sizes for main effects and interactions were calculated using partial eta squared (η^2^p), and interpreted as small (0.01 to 0.059) moderate (0.06 to 0.137), or large (>0.137). For pairwise comparisons, Hedges’ g was used to estimate effect size, classified as small (<0.3); medium (0.3–0.8); or large (>0.8). Additionally, Pearson’s correlation coefficients were computed to examine relationships between performance variables in the 5-H test and the DJ test. Statistical significance was set at *p* < 0.05. A power analysis was conducted using G*Power 3.1. Based on previous literature reporting large effect sizes for similar neuromuscular performance outcomes (Cohen’s d = 0.50 to 1.16) [14,22], and using f = 0.5 (equivalent to d ≈ 1.0) for a 2 × 2 repeated-measures ANOVA, a sample size of 12 participants provides sufficient power (80%) to detect large effects. The intraclass correlation coefficient (ICC) was calculated using a two-way mixed-effects model. The standard error of measurement (SEM) was derived using the formula SEM = SD × √(1 − ICC).

## 3. Results

### 3.1. Reliability Statistics of the 5-H Test

Table 1 presents the reliability statistics for the 5-H test, including total distance, reactive hopping index (RHI), and total time. The intraclass correlation coefficients (ICCs) indicated excellent reliability for total distance (ICC = 0.977), RHI (ICC = 0.968), and total time (ICC = 0.921). Coefficients of variation (CV) ranged from 1.8% (RHI) to 3.2% (total distance), suggesting excellent consistency. The percent standard errors of measurement (%SEM) were low across all variables, further supporting the reliability of the 5-H test.

### 3.2. Total Distance Covered in the Five Continuous Hop Test

**Figure 2:**
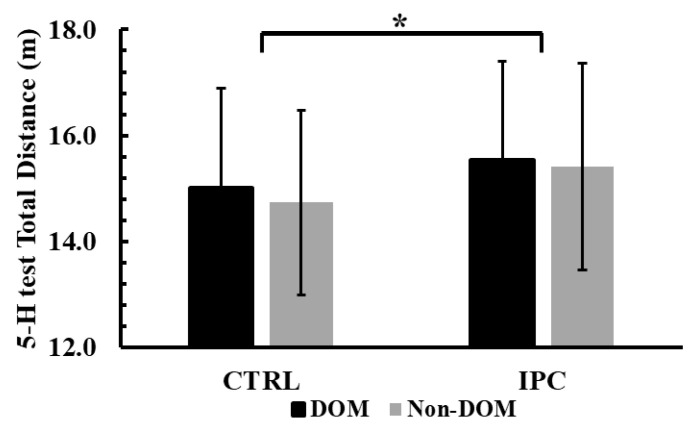
Total distance covered during the five-hop test (5-H) test across conditions (Ischemic Preconditioning (IPC) vs. Control (CTRL)) and legs (Dominant vs. Non-Dominant, DOM vs. Non-DOM). *: *p* < 0.001 from CTRL.

The two-way ANOVA (condition × leg dominance) revealed no significant interaction effect for total distance covered during the 5-H test (*p* = 0.73, η^2^p = 0.01). However, there was a significant main effect of condition (*p* < 0.001, η^2^p = 0.79). Tukey’s post hoc comparisons showed that the total distance covered in the IPC condition was +4.1% longer than in the control condition (15.48 ± 1.87 m vs. 14.87 ± 1.79 m or, *p* < 0.001, Hedges’ g = 0.32, see Figure 2).

### 3.3. Reactive Hop Index During the Five Continuous Hop Test

**Figure 3:**
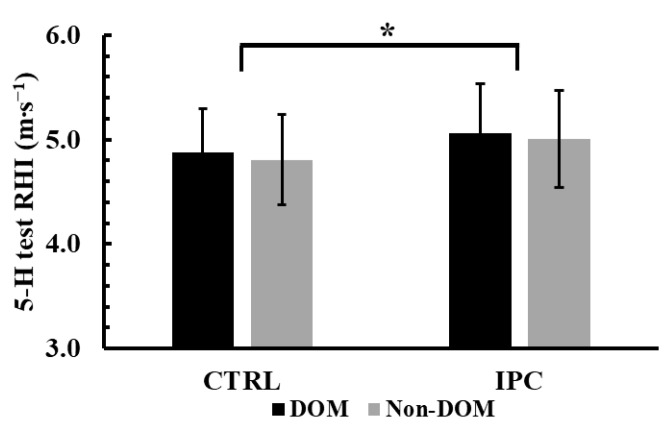
Reactive hopping index (RHI) across conditions (Ischemic Preconditioning (IPC) vs. Control (CTRL)) and legs (Dominant vs. Non-Dominant, DOM vs. Non-DOM). *: *p* < 0.001 different from the control conditions regardless of leg. *: *p* < 0.001 from CTRL.

The two-way ANOVA (condition × leg dominance) showed no significant interaction effect for the RHI during the 5-H test (*p* = 0.88, η^2^p = 0.002). However, a significant main effect of condition was observed (*p* < 0.001, η^2^p = 0.72). Tukey’s post hoc tests revealed that RHI was 3.9% higher in the IPC condition compared to the control in both legs (5.03 ± 0.46 m∙s^−1^ vs. 4.84 ± 0.42 m∙s^−1^, *p* < 0.001, Hedges’ g = 0.42, see Figure 3).

### 3.4. Total Time During the Five Continuous Hop Test

**Figure 4:**
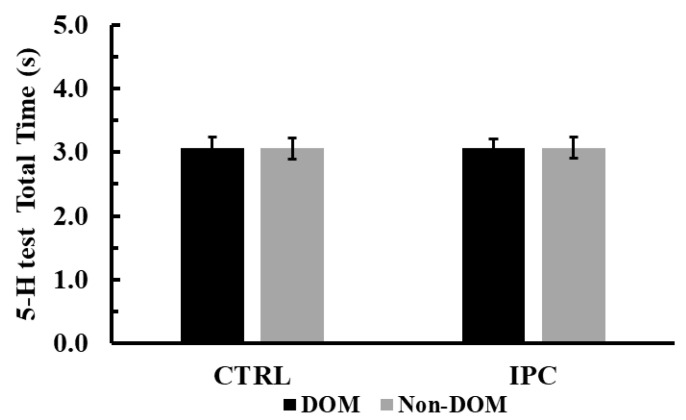
Total time during the 5-H test across conditions (Ischemic Preconditioning (IPC) vs. Control (CTRL)) and legs (Dominant vs. Non-Dominant, DOM vs. Non-DOM).

The two-way ANOVA (condition × leg dominance) revealed no significant interaction or main effects for total time during the 5-H test (*p* > 0.77, see Figure 4).

### 3.5. Reactive Strength Index During the Drop Jump Test

**Figure 5:**
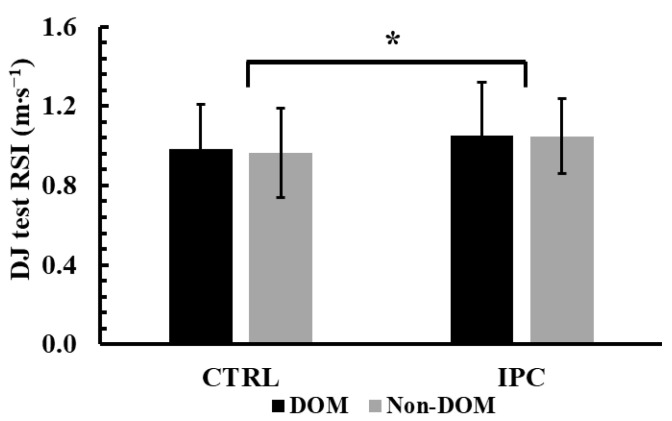
Reactive strength index (RSI) across conditions (Ischemic Preconditioning (IPC) vs. Control (CTRL)) and legs (Dominant vs. Non-Dominant, DOM vs. Non-DOM). *: *p* < 0.001 different from the control conditions regardless of leg.

The two-way ANOVA (condition × leg dominance) revealed no significant interaction effect for RSI during the DJ test (*p* = 0.71, η^2^p < 0.01). However, a significant main effect of condition was found (*p* < 0.001, η^2^p = 0.68). Tukey’s post hoc tests indicated that RSI was 7.8% higher in the IPC condition compared to the control in both legs (1.05 ± 0.23 m∙s^−1^ vs. 0.97 ± 0.22 m∙s^−1^, *p* < 0.001, Hedges’ g = 0.32, see Figure 5).

### 3.6. Jump Height During Drop Jump (DJ)

**Figure 6:**
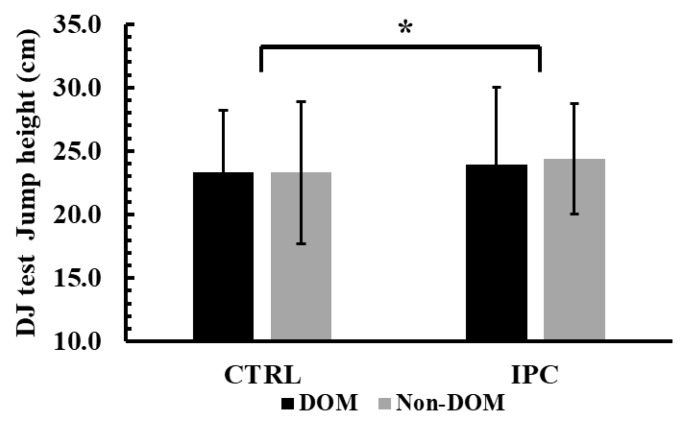
Jump height across conditions (Ischemic Preconditioning (IPC) vs. Control (CTRL)) and legs (Dominant vs. Non-Dominant, DOM vs. Non-DOM). *: *p* = 0.01 different from the control conditions regardless of leg.

The two-way ANOVA (condition × leg dominance) showed no significant interaction effect for jump height during the DJ test (*p* = 0.77, η^2^p = 0.001). However, a significant main effect of condition was observed (*p* = 0.01, η^2^p = 0.43). Tukey’s post hoc tests revealed that jump height during the DJ test was 3.6% greater in the IPC condition compared to the control in both legs (24.2 ± 5.2 cm vs. 23.3 ± 5.2 cm, *p* = 0.01, Hedges’ g = 0.16, see Figure 6).

### 3.7. Contact Time During Drop Jump (DJ)

**Figure 7:**
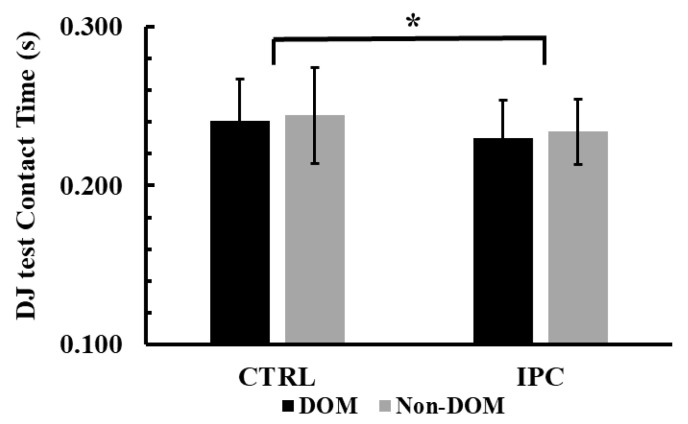
Contact time during the drop jump (DJ) test across conditions (Ischemic Preconditioning (IPC) vs. Control-CTRL) and legs (Dominant vs. Non-Dominant, DOM vs. Non-DOM). *: *p* = 0.01 significantly different from the control condition, regardless of leg.

The two-way ANOVA (condition × leg dominance) showed no significant interaction effect for contact time during the DJ test (*p* = 0.95, η^2^p = 0.0001). However, a significant main effect of condition was observed (*p* < 0.01, η^2^p = 0.51). Tukey’s post hoc comparisons showed that contact time during the DJ test was −4.4% shorter in the IPC condition compared to the control in both legs (0.232 ± 0.022 s vs. 0.242 ± 0.028 s, *p* = 0.01, Hedges’ g = 0.41, see Figure 7).

### 3.8. Relationships Between Variables

Table 2 presents the correlations among the assessed variables in the two tests. Total distance in the 5-H test was strongly correlated with RHI (r = 0.91–0.94; *p* < 0.05), DJ height (r = 0.82–0.85; *p* < 0.05), and DJ RSI (r = 0.83–0.88; *p* < 0.05) across conditions and legs. Additionally, RHI was significantly associated with both DJ RSI (r = 0.71–0.85; *p* < 0.05), and DJ height (r = 0.66–0.79; *p* < 0.05).

## 4. Discussion

The main finding of the present study was that IPC significantly improved performance in both the 5-H test and the DJ test, irrespective of leg dominance. Specifically, IPC led to greater total distance and a higher RHI in the 5-H test, as well as increased RSI, greater jump height, and reduced contact time during the DJ test, compared to the control condition. These performance improvements were consistent across both dominant and non-dominant legs, with effect sizes ranging from small to moderate. Importantly, these changes should be interpreted in the context of the test–retest reliability of the key variables. The 5-H test demonstrated excellent reliability for total distance (ICC = 0.966) and RHI (ICC = 0.920), and good reliability for total time (ICC = 0.850), with low SEM and acceptable CV values (e.g., CV < 10%). These results support the consistency and sensitivity of the test in detecting true performance changes, reducing the likelihood that observed differences were due to measurement error. Strong and significant correlations were also observed between total distance in the 5-H test and key performance metrics such as RHI, DJ RSI, and DJ height, suggesting that the 5-H test captures essential elements of reactive strength and explosive power. Another noteworthy finding was the absence of any interaction between condition and leg dominance across all outcome variables, indicating that the IPC protocol had a uniform effect on both legs. This uniformity supports the applicability of IPC as a practical preconditioning strategy for both symmetric and asymmetric athletes. Collectively, these results suggest that IPC may acutely enhance horizontal and vertical jumping performance, likely through improvements in neuromuscular explosiveness.

Our results are in line with previous research reporting acute ergogenic effects of IPC on explosive lower-body movements, such as vertical jumps. Doma et al. (2020) and Ricart Luna et al. (2022) demonstrated improved CMJ performance following IPC [14,22], while Beaven et al. (2012) also observed a positive effect of IPC on concentric and eccentric force, in both CMJs and squat jumps (SJs) [40]. Although these studies typically employed more prolonged protocols with multiple ischemia–reperfusion cycles (2–4 × 3–5 min IPC, 5 min reperfusion), the current findings extend this knowledge by demonstrating that even a single IPC cycle can elicit significant performance benefits. This also aligns with the results of Salagas et al. (2022), who showed increased performance during repeated sets of upper body resistance training, following short-duration IPC application [5]. Unlike most prior studies, we also included a horizontal reactive strength task (5-H test), which simulates sport-specific movements, particularly for jumpers. To our knowledge, this is the first study to report IPC-induced improvements in multi-hop horizontal performance, which broadens the understanding of IPC’s application beyond vertical metrics.

On the other hand, while the present study found performance-enhancing effects in explosive short-duration tasks involving both vertical and horizontal reactive strength, some meta-analyses and systematic reviews have reported inconsistent or negligible effects of IPC on similar high-intensity anaerobic activities, such as sprints and maximal acceleration tasks [10,12]. This discrepancy may relate to the task-specific nature of IPC’s effectiveness. Short-distance sprinting relies heavily on maximal acceleration and brief explosive efforts, with ground contact times often below 150 milliseconds during the initial steps [41,42]. Such limited durations may not provide sufficient time for the physiological mechanisms facilitated by IPC—such as improved muscle perfusion, enhanced motor unit recruitment, or increased neuromuscular drive—to exert a meaningful effect on performance [6,10,12]. In contrast, repeated hopping and DJ tasks, like those used in the current study, involve longer stretch-shortening cycles and brief but sustained reactive efforts, potentially offering a more favourable physiological window for IPC to manifest its benefits. Notably, studies reporting null effects of IPC [12], often used cycling sprints or single linear runs, while in our protocol, both the 5-H and DJ tests involved multi-joint, unilateral, and sport-specific explosive tasks, which may be more sensitive to neuromuscular potentiation.

Performance improvements in the current study may be attributed to various potential physiological mechanisms resulting from IPC application. The observed increases in DJ variables, along with the greater total distance and horizontal velocity in the 5-H test, suggest enhanced neuromuscular performance following IPC. One likely contributing factor is the attenuation of Type III and IV muscle afferent feedback that has been reported to result from IPC [13,15,16,17], which may have reduced inhibitory signals to the central nervous system and allowed for greater motor unit recruitment during explosive movements. This mechanism is particularly relevant to tasks with high stretch-shortening cycle (SSC) demands, such as DJs and repeated hopping, wherein rapid transitions between eccentric and concentric phases are essential. Additionally, improved muscle perfusion during the reperfusion period—possibly mediated by increased nitric oxide (NO) availability [13] —may have enhanced oxygen delivery and calcium signaling in the working muscles, thus improving force and power generation during the tests used [43]. Last, the recently proposed similarity between IPC and the Post-Activation Performance Enhancement (PAPE) effect may help explain the acute performance improvements observed in both the DJ and 5-H tasks. In line with our findings, Salagas et al. (2022) reported enhanced performance following IPC in the bench press exercise, reflected in both an increased number of repetitions and higher movement velocity—an outcome indicative of improved dynamic and explosive strength [5].

These findings have direct implications for both coaches and athletes, particularly in track and field. A single, time-efficient IPC cycle could be implemented during warm-up routines to acutely enhance both vertical and horizontal power outputs without the logistical and time demands of multi-cycle protocols. This approach may be especially useful in competition settings, where time constraints and performance optimization are critical. However, further studies are required to examine if this IPC protocol can be applied on both legs simultaneously, and the duration of the positive effects of IPC after a single 5 min protocol is applied. The fact that performance improvements occurred regardless of leg dominance further supports the general applicability of this protocol. Additionally, the strong correlations observed between DJ performance (RSI, jump height) and the 5-H test outcomes (RHI, total distance) suggest that a simple field test such as the 5-H test DJs may serve as a practical field-based proxy to monitor horizontal and vertical reactive strength indices.

This study has some limitations that should be acknowledged. First, the sample size was modest and included both male and female athletes, which may introduce variability despite the repeated-measures design. Second, the study assessed only acute effects; thus, the long-term impact or repeated use of IPC on performance remains unclear. Third, due to the unilateral design, some cross-education effects or between-leg interactions cannot be entirely ruled out. Finally, although the protocol was time-efficient, manual cuff inflation might introduce variability in ischemia timing; the use of automated systems in future research could address this limitation.

## 5. Conclusions

In conclusion, this study demonstrates that a single, short-duration IPC cycle can significantly enhance both vertical and horizontal jumping performance in trained jumpers. These findings support the use of IPC as a practical, time-efficient warm-up strategy to acutely enhance neuromuscular performance. The strong relationship between drop jump metrics and horizontal hopping performance also highlights the potential utility of the 5-H test to monitor training adaptations in reactive strength. Importantly, these effects were consistent across both dominant and non-dominant legs, indicating that the efficacy of IPC is not influenced by leg dominance. Finally, the high reliability of the performance metrics used (as indicated by ICC, SEM, and CV values) reinforces the robustness of our findings and suggests that the improvements observed are likely to reflect genuine physiological adaptations rather than random variation or measurement error.

## Figures and Tables

**Table 1:** Reliability statistics for the 5-H test.

Statistical Metrics	5-H Test Total Distance (m)	5-H Test RHI (m∙s^−1^)	5-H Test Total Time (s)
**ICC**	0.977	0.968	0.921
**(lower–upper)**	(0.945–0.993)	(0.923–0.990)	(0.811–0.975)
**CV (%)**	3.2%	1.8%	2.7%
**%SEM**	1.8%	1.6%	1.6%

ICC: intraclass correlation coefficient; CV: coefficient of variation; %SEM: standard error of measurement (%).

**Table 2:** Correlations between measured variables across different conditions and legs.

		5-H Test Total Distance				5-H Test RHI			
		IPC-DOM	CTRL-DOM	IPC-Non-DOM	CTRL-Non-DOM	IPC-DOM	CTRL-DOM	IPC-Non-DOM	CTRL-Non-DOM
**5-H test RHI**	**IPC-DOM**	0.94 **							
	**CTRL-DOM**		0.92 **						
	**IPC-Non-DOM**			0.92 **					
	**CTRL-Non DOM**				0.91 **				
**DJ height**	**IPC-DOM**	0.83 **				0.72 **			
	**CTRL-DOM**		0.85 **				0.76 **		
	**IPC-Non-DOM**			0.82 **				0.79 **	
	**CTRL-Non DOM**				0.82 **				0.66 *
**DJ RSI**	**IPC-DOM**	0.88 **				0.79 **			
	**CTRL-DOM**		0.88 **				0.84 **		
	**IPC-Non-DOM**			0.83 **				0.85 **	
	**CTRL-Non DOM**				0.85 **				0.71 **

RSI: reactive strength index; RHI: reactive hopping index; CTRL: control; IPC: ischemic preconditioning; DOM: dominant leg; Non-DOM: non-dominant leg;. *: *p* < 0.05, **: *p* < 0.01.

## Data Availability

The data are available upon request to the corresponding author.
